# Transcriptome and Metabolome Analyses Provide Insights into the Occurrence of Peel Roughing Disorder on Satsuma Mandarin (*Citrus unshiu* Marc.) Fruit

**DOI:** 10.3389/fpls.2017.01907

**Published:** 2017-11-07

**Authors:** Xiao-Peng Lu, Fei-Fei Li, Jiang Xiong, Xiong-Jun Cao, Xiao-Chuan Ma, Zi-Mu Zhang, Shang-Yin Cao, Shen-Xi Xie

**Affiliations:** ^1^Horticulture Department, College of Horticulture and Landscape, Hunan Agricultural University, Changsha, China; ^2^National Centre for Citrus Improvement, Changsha, China; ^3^Institute of Horticulture, Hunan Academy of Agricultural Science, Changsha, China; ^4^Zhengzhou Fruit Research Institute, Chinese Academy of Agricultural Sciences, Zhengzhou, China

**Keywords:** satsuma mandarin, roughing disorder, GA, starch, mineral nutrients, RNA-sequencing, metabolome

## Abstract

Roughing disorder (RD) is a significant quality barrier in citrus fruit, prevalent on easy-peeling mandarins. As RD is not yet well-understood, this study aimed to examine the changes and synergic molecular processes involved in peel RD. Peel with RD was induced by severely defruiting Satsuma mandarin trees. Morphology observations, RNA-sequencing, and targeted and untargeted metabolic analyses were conducted. The results showed that the primary metabolites of sugars, organic acids and amino acids are dramatically changed in RD peel. The RD peel was always characterized by higher magnesium content during development. Comparative transcriptome profiling was performed for CK and RD peels at 30, 80, and 170 days after full bloom (DAFB) which represented fruit at cell division stage, cell enlargement stage and fruit maturity stage, respectively. Physiological and molecular biological evidence suggested that the month after full bloom is a crucial stage for RD initiation. A total of 4,855 differentially expressed genes (DEGs) in RD peel, relative to CK peel were detected at cell division stage, about 2 to 4-fold more than other stages had. Among the differentially expressed transcription factors, the bHLH family were affected most by RD, and six bHLH transcription factors functionally involved in GA metabolism were assessed to associate with RD occurrence. Gene set enrichment analysis suggested that RD significantly altered starch and GA metabolism in peel. Higher starch content and hydrolysed chain status were found in RD peel at cell division stage. RD occurrence on the peel was influenced significantly by GA, especially abundant GA before July. These changes may mean a significant alteration in sink strength of RD peel. The findings of this study provide insights into the emergence, development and molecular mechanisms of RD.

## Introduction

The Satsuma mandarin (*Citrus unshiu* Marc.) is an important easy-peeling mandarin around the world, and Asian countries including China, Japan and Korea are the largest producers. In China, the Satsuma mandarin is one of two important easy-peeling mandarins, and makes up more than thirty per cent of total citrus production each year. Although the Satsuma mandarin is popular among world consumers, farmers in traditional growing areas are facing serious pressures, such as low prices due to large yields, fruit quality decline, and faster cultivar renewal. Of these, fruit quality problems are the most significant and receive more attention from producers and consumers. Roughing disorder (RD), also termed rough fruit, rough peel disorder (Erner et al., 1975, 1976; Liu, 2012), peel roughness (Liu et al., 1988), and rind roughness (Kubo and Hiratsuka, 1998, 1999) is a common and typical quality barrier in Satsuma mandarin fruit. RD is characterized by excessively thick, rough peel and large fruit size (Erner et al., 1976). It significantly impairs the commodity value of Satsuma mandarin fruit, but the molecular mechanisms involved are still not clear.

RD of Satsuma mandarin fruit occurs through a complicated process, and is influenced by hormones, soil, air humidity, and rootstock (Erner et al., 1976). Two major factors often lead to RD in Satsuma mandarin plants. The first factor is low fruit load. Citrus trees in the primary fruiting stage usually produce few fruit, most of which are characterized by RD. In addition, a heavy fruit load (ON-Crop) in 1 year inhibits the return bloom in the following year (Monselise and Goldschmidt, 1982; Shalom et al., 2014). The second year is characterized by a low yield (OFF-Crop) in which fruit with RD are predominant. The second factor that induces RD is the bearing angle. On a Satsuma mandarin tree, upward fruit frequently develop RD, but sideward fruit are smoother. A marked difference between them can be observed from mid-July (Kubo and Hiratsuka, 1998). The growth condition of the bearing basal shoot is also associated with RD. Generally, the bearing basal shoot is a spring and leafy shoot, usually shorter than 6 cm and thicker than 0.4–0.6 cm (Liu, 2012). Unlike fruit puffiness, which appears near or during the maturity stage, RD could be commonly observed during Satsuma mandarin fruit development. Satsuma mandarin fruit on low-crop-load trees exhibit RD symptoms in mid-June, showing higher fruit weight, bigger epidermal size, and hypodermal and parenchymal cells in the peel (Kubo and Hiratsuka, 1999). Because there is an increase in cell layer and cell diameter 7–28 days after full bloom (DAFB), this period has been identified as key in RD development (Liu, 2012).

A histological comparison of normal and RD fruit suggested that vigorous oil gland and hypodermal tissues contribute to RD in Satsuma mandarin fruit (Kubo and Hiratsuka, 1999). Another histological comparison of two peel types showed that peel thickness increment in RD fruit depended on the number of cell layers but not cell size (Liu, 2012). Hormones are believed to be another important factor behind RD in Satsuma mandarin. It is believed that exogenous GA_3_ application at an early fruit development stage induces RD (Liu et al., 1987). Upward fruit on “Okitsu wase” mandarin (*C. unshiu* Marc.) trees tending to have RD had higher GA content than sideward fruit with smooth peel. Exogenous applications of different hormones suggested that GA_3_ and BA treatments in late June significantly induced RD whereas others did not (Kubo and Hiratsuka, 1998, 2000). “Owari” mandarin (*C. unshiu* Marc.) fruit enlarged significantly with 2,4-DP, but fruit peel thickness did not (Agusti et al., 1994).

RD probably occurs in situations where source/sink ratio is excessive, but the mechanism of initial RD occurrence in Satsuma mandarin is still unclear. RD involves changes to fruit size, peel, segment membrane, juice sac, juice and so on, among which peel RD is the most obvious and typical phenotype. Although morphological changes have been documented, metabolic profiling of RD peel has not been investigated. The untargeted metabolome is appropriate to this study because metabolic fluxes might be directly related to peel RD. Thus, in this study we first aimed to reveal the overall mechanism of peel RD occurrence through comprehensive exterior and interior phenotypic characterization, along with genome-wide gene expression throughout RD peel development. Finally, metabolites, mineral nutrients, synergic pathways and genes correlated with the development of peel RD were identified. The results presented help clarify previously fragmentary knowledge, and provide further insight into the genetic and physiological basis of peel roughing, an important *Citrus* disorder.

## Materials and methods

### Plant materials and sampling

Eight-year-old “Yamashitabeni wase” Satsuma mandarin (*C. unshiu* Marc.) grafted on trifoliate orange [*Poncitrus trifoliata* (L.) Raf.] rootstocks were used in this study. Fifteen healthy and approximately uniform trees were selected for the experiment and fruit with RD were induced via severe fruit removal. At fruit set stage in spring, 10 trees were defruited, with 15 upward fruits left on each tree, while the other five trees without fruit removal served as the control (CK). For CK and treatment, fruit was randomly harvested at 20, 30, 40, 50, 60, 80, 110, 140, and 170 DAFB. For each CK sampling, 30 fruits from three CK trees with 10 for each tree were sampled. For treatment sampling, 21 fruits from three treated trees with seven fruits for each tree were sampled at 20, 30, and 40 DAFB, 15 fruits from three treated trees with five fruits for each tree at 50, 60, and 80 DAFB and nine fruits from at least three treated trees at 110, 140, and 170 DAFB were collected. At each sampling date, fruit growth was estimated by measuring transverse diameter of 30 fruits growing on the trees for CK and 30 fruits growing on the trees or all the remaining fruits if there were < 30 for treatment. Peel thickness was measured on all fruits individually. For each of the triplicate fruit groups mentioned above, the peel was isolated, immediately frozen in liquid nitrogen, and stored at −80°C for further analyses; the fruit pulp was stored at −40°C for targeted metabolites analyses.

### Morphological observation for the peel using SEM

CK and RD peels isolated from fruit at 30, 80, and 170 DAFB, representing fruit at cell division stage, cell enlargement stage and fruit maturity stage, respectively were used for SEM. At each of three sampling dates, six 5 × 5 mm peel squares from three fruits were collected from CK peel and RD peel, respectively. The samples were dehydrated using a graded ethanol series (50, 70, and 100%), critical-point dried, mounted on copper stubs and gold sputtered. The samples were examined under a JSM-6380LV scanning electron microscope (SEM, Jeol, Tokyo, Japan). Both peel surface and cross section were observed by SEM and representative images were selected.

### Untargeted and targeted metabolites analyses

To analyse untargeted metabolites in RD peel, another five “Yamashitabeni wase” Satsuma mandarin trees, each of which typically had both smooth (CK) fruits and RD fruits were chosen. In each of five trees, three downward CK fruits and three upward RD fruits were collected and had peel isolated at 170 DAFB. Peel samples from three fruits on one tree were mixed as a replicate with a total of five replicates for both CK and RD. The untargeted metabolic profiling was performed according to published methods with minor modification (Osorio et al., 2012). An Agilent 5975C MSD mass spectrometer (MS, Agilent Technologies, Palo Alto, CA, USA) coupled to an Agilent 7890A gas chromatograph (GC) system was used. Ten micrograms of ground and freeze-dried samples were used for metabolite extraction with 1 mL pre-chilled solvent mixture (Acetonitrile: Isopropanol: Water, 3: 3: 2), and underwent ultrasonication for 32s in ice-water mixture. Samples were then centrifuged for 10 min at 13,000 rpm and 4°C and supernatant was collected. Eight hundred microliter aliquots of supernatant plus 8 μL of 3 mg/mL Myristic acid-*d*_27_ were concentrated to complete dryness with nitrogen blowing. The residue was dissolved in 20 μL of 40 mg/mL methoxyamine hydrochloride/pyridine, and incubated for 90 min at 30°C. Samples were then treated with 90 μL N-methyl-N-(trimethylsilyl) trifluoroacetamide (1% trimethylchlorosilane) for 30 min at 37°C. After a second centrifugation at 12,000 rpm for 2 min, the supernatant was analyzed. Relative concentrations of the metabolites were determined by peak area (mm^2^) and the mass spectra were then compared to known and commercially available mass spectral libraries.

For targeted amino acid determination in CK and RD peels, triplicate samples out of five used in GC-MS were chosen for analysis. Peels were dried at 75°C for 3 days and ground immediately in a disintegrator (FW100, TAISITE, Tianjin, China). One gram powder samples were analyzed using the published method with a HITACHIL-8900 amino acid analyser (Hitachi, Wako, Japan) (Lu et al., 2016). To measure the targeted starch, organic acids and sugars, the isolated and frozen CK and RD peels at 40, 80, 110, 140, and 170 DAFB were used. The starch content was determined according to the published method with slight modification (Xu et al., 1998). One hundred milligram peel samples were ground in 80% alcohol and centrifuged at 4,000 rpm for 2 min. The suspension was collected and the residue was extracted another three times with 80% (m/v) Ca(NO_3_)_2_ at 100°C (5 mL each, 10 min per extraction). The extract was added to a final volume of 20 mL with 80% Ca(NO_3_)_2_. One milliliter extract solution was diluted using 1 mL 80% Ca(NO_3_)_2_, colored with 100 μL 0.1 M I_2_-KI and then absorption determined at 620 nm using a spectrophotometer (UV-1800, Shimadzu, Kyoto, Japan). The absolute amount of starch was determined by comparison with calibration standard curve and calculated based on peel fresh weight. Organic acid and sugar components were measured using high-performance liquid chromatography (HPLC) (LC-20A, Shimadzu, Kyoto, Japan) according to the published method (Lu et al., 2016). Organic acid and sugar were also determined in pulp using samples stored at −40°C. Triplicate tissue samples were analyzed for the above assays.

### Mineral nutrient analyses

For difference-screening analyses of macro- and micro-minerals between CK and RD peels, the same samples from targeted amino acid analyses were used. For targeted mineral nutrient, peel samples collected at 30, 80, and 110 DAFB, as described in the Plant Materials and Sampling section, were chosen to test the reliable content-difference between CK and RD peels. The peel samples were dried at 75°C for 3 days and ground immediately in a disintegrator (FW100, TAISITE, Tianjin, China). In assay, N and P were extracted and measured according to published methods (Bao, 2000). Peel K, Ca, Mg, Mn, Cu, and Zn were extracted with 1 M HCl and assayed using atomic absorption spectrophotometry. B was measured using the curcumin method after ashing the sample at 500°C for 5 h and dissolving in 0.1 M HCl (Li et al., 2015).

### Transcriptome sequencing and analyses

According to citrus fruit size and peel thickness development, 30, 80, and 170 DAFB were chosen to represent the fruit cell division stage, cell enlargement stage and fruit maturity stages respectively. Six libraries were constructed for transcriptome sequencing and named peel for control at 30 DAFB (CK30), peel for control at 80 DAFB (CK80), peel for control at 170 DAFB (CK170), peel with RD at 30 DAFB (RD30), peel with RD at 80 DAFB (RD80), and peel with RD at 170 DAFB (RD170). For each library construction, peels subsampled from triplicate peel samples described in Plant Materials and Sampling were mixed to produce a pool. One gram of peel was used for total RNA isolation using TRIzol reagent (TaKaRa, Dalian, China) according to the manufacturer's instructions, and the mRNA was enriched using magnetic oligo (dT) beads. Preparation of the paired-end libraries and sequencing was performed following standard Illumina methods and protocols. The cDNA libraries were sequenced on an Illumina Hiseq2000 platform and 100 bp single-end reads were generated.

Clean reads were obtained by removing reads containing adapter, ploy-N and low quality sequence from raw data, and were aligned to the *Citrus sinensis* genome (Xu et al., 2013) using Bowtie (Langmead et al., 2009) and BWA (Mortazavi et al., 2008). To estimate gene expression levels, fragments per kilobase per million mapped reads (FPKM) of each gene were calculated (Li and Dewey, 2011). Differentially expressed genes (DEGs) in pairwise comparisons were then identified (Audic and Claverie, 1997). All the statistical results for multiple testing were corrected with the Benjamini-Hochberg false discovery rate (FDR < 0.001) (Benjamini and Yekutieli, 2001). Sequence expressions were deemed to be significantly different if FDR < 0.001 and there was at least a 2-fold change (>1 or <-1 in log^2^ ratio value) in FPKM between two libraries. Gene Ontology (GO) annotation was performed using Blast2 GO software (Conesa et al., 2005). Finally, DEGs were enriched in GO and Kyoto Encyclopedia of Genes and Genomes (KEGG) databases so as to identify the changes in biological functions and metabolism pathways. In addition, all DEGs were aligned in a plant transcription factor database (Pérezrodríguez et al., 2009) using HMMER (Finn et al., 2015) to screen the transcription factors.

### Quantitative real-time PCR analyses

Total RNA was isolated from the frozen peel following the method described above. Roughly 1.0 μg total RNA was used for first-strand cDNA synthesis using the iSCRIPT cDNA synthesis kit (Bio-Rad) following the manufacturer's instructions. Specific primers were designed from the selected gene sequences using Primer Express Version 3.0 (Applied Biosystems, CA, USA) and the primer sequences are given in Table S1. Quantitative RT-PCR was performed according to previous reports (Yan et al., 2012; Lu et al., 2016). Samples for qRT-PCR were run in three biological replicates with three technical replicates.

### Hormone treatment experiment

In the same plot described in plant material, six healthy and approximately uniform “Yamashitabeni wase” trees were chosen for the hormone treatment experiment. Three trees grown normally served as the control (CK). For IAA, CTK, GA_3_ and mixture (IAA+CTK+GA_3_) treatments, 20 representative fruits on each of another three trees were treated with the corresponding solutions. Aqueous solutions for IAA, CTK, GA_3_, and mixture treatments contained 50 mg·L^−1^ IAA, 3 mg·L^−1^ CTK, 50 mg·L^−1^ GA_3_, and the mixture with their own concentration respectively. Triton X-100 (0.05%) was added to each solution as a surfactant. At 40 DAFB, 20 representative fruit on each tree were soaked in treatment solution for 1 min. This was repeated again at 50 DAFB. At 85 and 115 DAFB in CK and treatments, 10 fruit from each treated tree for a total of 30 fruit per phenotype were collected. The collection of fruit size and peel thickness data, isolation of peel and pulp, determination of organic acid and sugar were all identical to the methods described above.

### Statistical analyses

Five individual biological replicates were used for GC-MS analyses and data were statistically analyzed using Student's *t*-test (*P* < 0.05). Three biological replicates were used in the organic acid, sugar, amino acid, and mineral nutrient determination and data were analyzed statistically using Duncan's multiple range test in an ANOVA program of SAS (Cary, NC, USA) at *P* < 0.05. Due to 30 fruits for CK in each sampling but 9 to 21 fruits for RD depending on sampling, Duncan's multiple range test for unequal replication in an ANOVA program at *P* < 0.05 was employed for the statistical analyses of data in fruit size and peel thickness.

## Results

### Characteristics of satsuma mandarin fruit with RD

**Figure 1:**
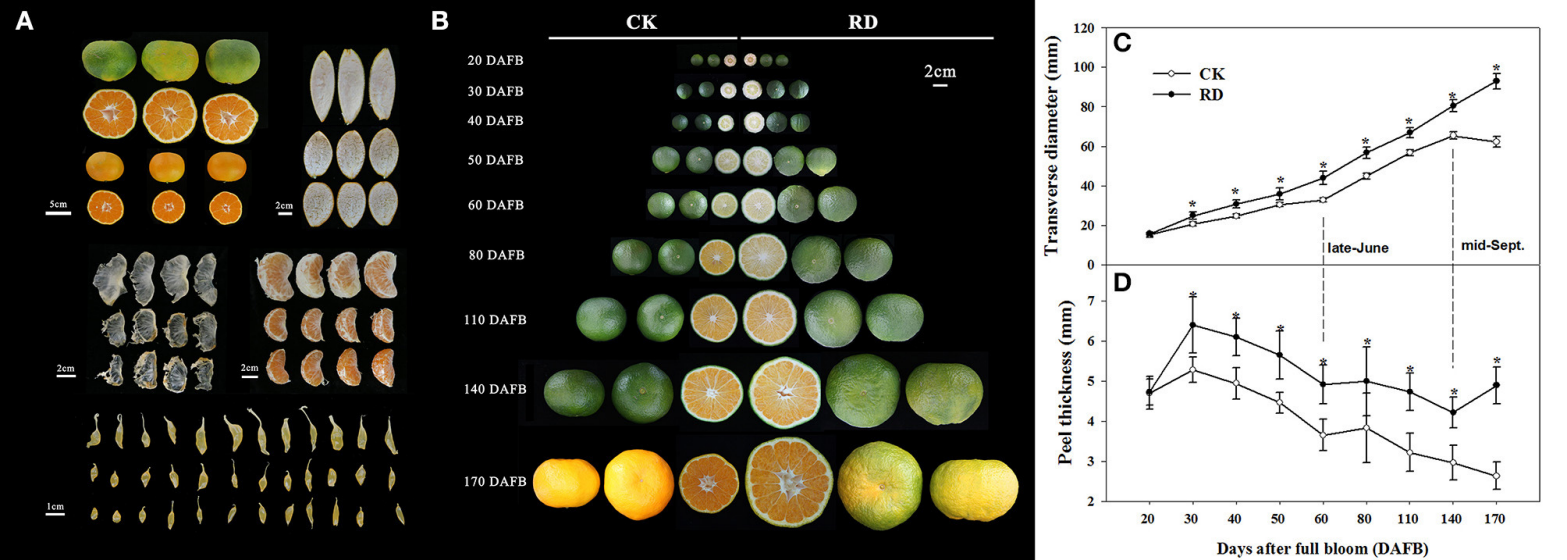
Characteristics and development of Satsuma mandarin fruit with roughing disorder (RD). **(A)** The exterior and interior characteristics of RD fruit. **(B)** Fruit development for CK and RD fruits. Fruit size **(C)** and peel thickness **(D)** changes throughout fruit development. Asterisk means significant difference at *P* < 0.05.

Relative to CK fruit, RD fruit have significantly different interior and exterior characteristics. Rough fruit surfaces, bigger fruit sizes and delayed fruit degreening are noticeable in RD fruit. Furthermore, thicker peels, enlarged fruit segments, and bigger juice sacs all contribute to the increased size of RD fruit. In RD fruit, the segment membrane and the tail of juice sacs are whiter and more indistinct, suggesting severe lignification (Figure 1A). RD fruit showed significant differences in development patterns compared to CK fruit. Although both types of fruit shared similar characteristics at 20 DAFB, RD fruit were larger than CK fruit at 30DAFB and had a rough surface. At 170 DAFB, RD fruit was developing color whereas CK fruit had colored completely (Figure 1B). Satsuma mandarin fruit develop through cell division, cell enlargement, and maturity stages, during which fruit size changes in distinct patterns (Bain, 1958; Kubo and Hiratsuka, 1998). In this study, CK fruit clearly followed that process: cell division occurred from flowering to 60 DAFB (late-June), cell enlargement from 60 to 140 DAFB (mid-September), and fruit maturation from 140 to 170 DAFB (mid-October). In contrast, RD fruit size constantly increased throughout fruit development, differing from the classical development pattern. RD fruit always had thicker peel than CK fruit, but both followed the three-stage growth pattern (Figure 1C). Peel thickness of CK and RD fruit rapidly peaked at 30 DAFB and then decreased until 60 DAFB. A plateau phase in peel thickness growth occurred from 60 to 80 DAFB. After that, peel thickness continued to decrease in CK fruit, but RD peel thickness was stable from 140 to 170 DAFB (Figure 1D).

### Ultrastructure alterations of RD peel during development

**Figure 2:**
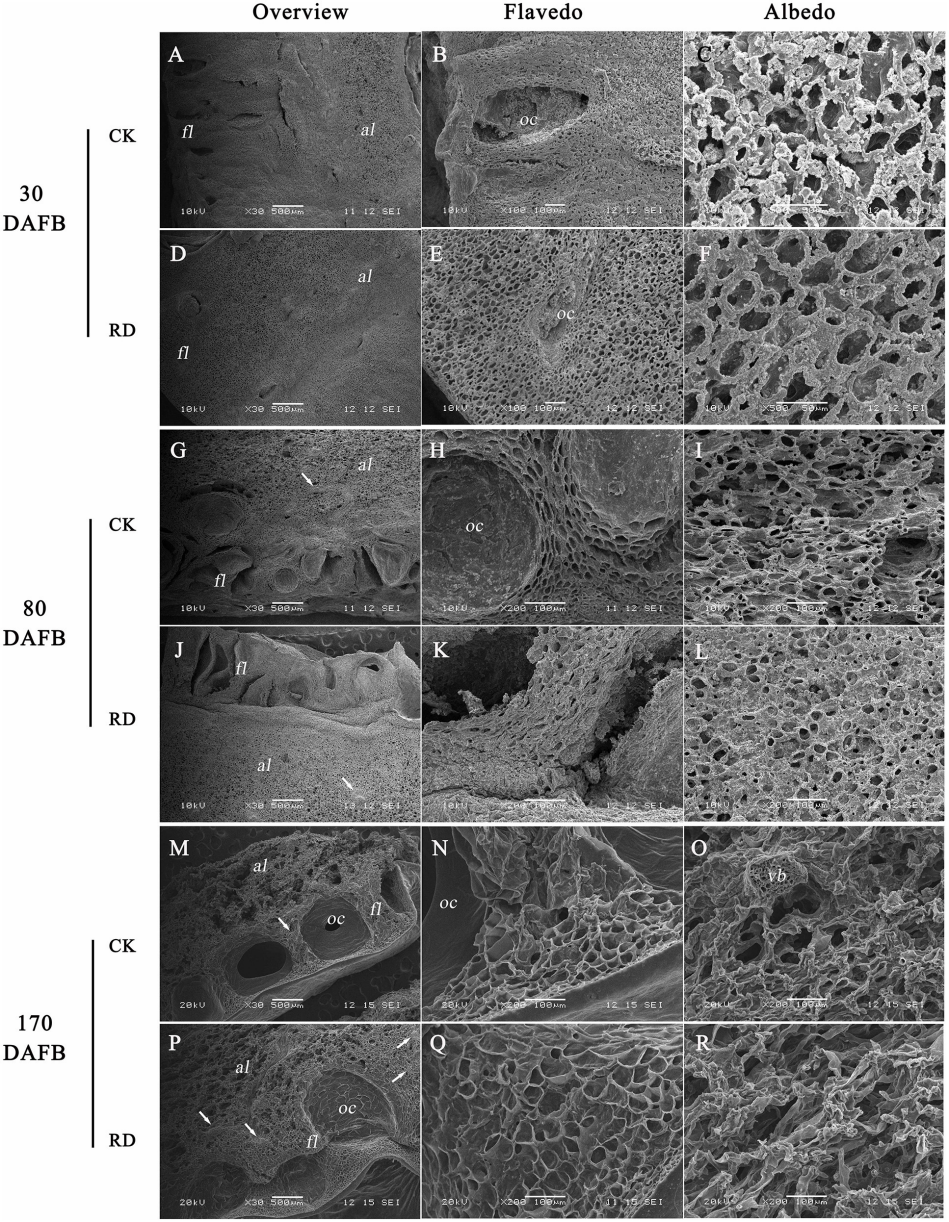
Development of peel RD at three fruit development stages. The overview **(A,D)**, flavedo **(B,E)** and albedo **(C,F)** of CK and RD peels at 30 DAFB. The overview **(G,J)**, flavedo **(H,K)** and albedo **(I,L)** of CK and RD peels at 80 DAFB. The overview **(M,P)**, flavedo **(N,Q)** and albedo **(O,R)** of CK and RD peels at 170 DAFB. The white arrow shows the vascular bundle in the peel. *fl, flavedo; al, albedo; oc, oil cell; vb, vascular bundle*.

Due to the significant changes in RD peel, scanning electron microscopy was used to study the ultrastructure alterations. Both peels were examined at 30, 80, and 170 DAFB (representing fruit at cell division, cell enlargement and maturity stage) to provide insights into the development of RD peel. At 30 DAFB, flavedo and albedo (which compose citrus peel) could not be clearly distinguished. At this stage the peels mainly comprised parenchymatic cells and few oil cells. Compared to CK, RD, peel had much greater thickness, more cells and lower oil cell density (Figures 2A,D). Furthermore, at this stage RD peel had bigger cell sizes, more clearly shaped parenchymatic cells, bigger intercellular spaces and thinner cell arrangement than CK peel (Figure 2B,C,E,F). Greater polysaccharide effluence was observed in CK peel than in RD peel (Figures 2C,F). At 80 DAFB, the flavedo and albedo of RD peel were thicker than those of CK peel and vascular bundles were observed. CK peel clearly had regular oil cells while flavedo was irregular in RD peel (Figures 2G,J). CK flavedo had spherical oil cells with smooth inner walls, but the oil cells in RD flavedo took non-identical sizes and had rough inner walls. In addition, normal parenchymatic cells layered regularly in CK flavedo, but the cell wall of these cells were significantly thickened in RD flavedo (Figures 2H,K). Lower cell density and larger intercellular space increased in RD albedo at 80 DAFB, consistent with the increase in RD peel (Figures 2I,L). CK and RD fruit exhibited relatively thin peel at 170 DAFB, although RD peel was still thicker than CK. CK flavedo remained spherical and featured small oil cells still characterized by smooth inner walls, but in the bigger oil cells in RD flavedo, a laminated structure on the rough inner-wall was found. Moreover, flavedo in RD peel had more epidermal cell layers and bigger cell size relative to CK (Figures 2N,Q). Vascular bundles in albedo were clearer in both peels at 170 DAFB, but the amount appeared greater in RD albedo (Figures 2M,P). At fruit maturation, flocculent structures filled albedo instead of regular cells. However, flocculent structures were much more loosely arranged and had thicker cell walls in RD albedo than in CK albedo (Figures 2O,R).

### Changes of metabolites and mineral nutrients in RD peel

**Figure 3:**
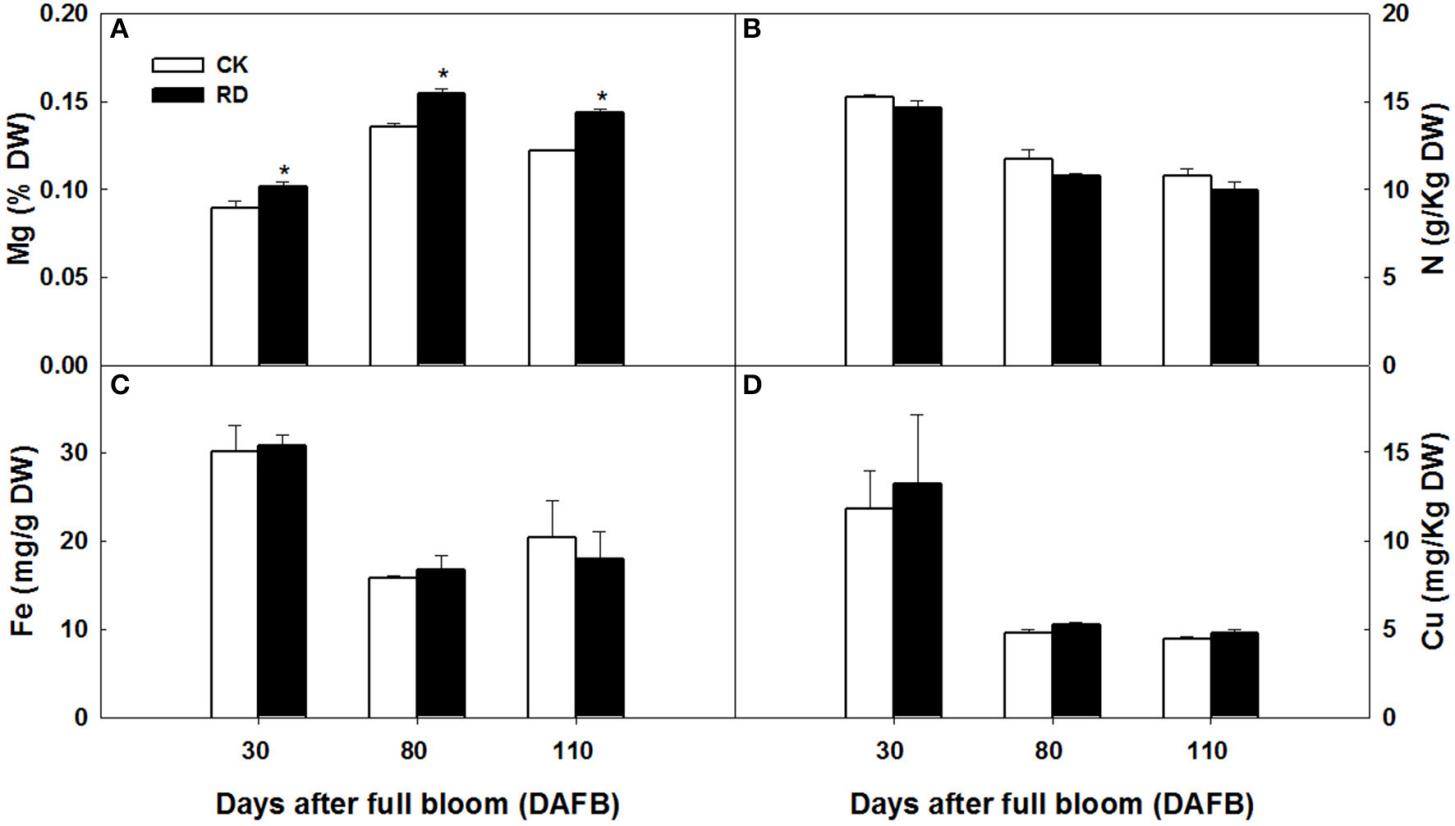
Mg **(A)**, N **(B)**, Fe **(C)** and Cu **(D)** content in CK and RD peels during development. Asterisk means significant difference at *P* < 0.05.

Using GC-MS, an untargeted metabolomic analyses was conducted to identify the metabolites associated with peel RD. Because the typical and complete RD occurs at fruit maturity stage, the peels at 170DAFB were chosen for comparison. As the relative concentrations of the metabolites were estimated by peak area, 855 metabolites were identified in both CK and RD peels. According to >1.2 or < 0.8 in RD/CK ratio and statistical analyses, 30 metabolites were significantly different across CK and RD peels. These metabolites were classified as sugars, organic acids, amino acids and derivatives, alcohols, heterocyclic compounds, amines, and two other compounds (Table 1). Of these classifications, the main differences were found in sugars, organic acids, amino acids, and derivatives. Notably, most amino acids and derivatives were significantly higher in RD peel than in CK peel, and citrulline, beta-homoserine, and glutamine were 4.80-, 5.51-, and 8.43-folds higher in RD peel, respectively. In further targeted amino acid analyses, 13 of 17 amino acids were significantly higher in RD peel (Table 2). Three amino acids were lower in RD peel and Arg was not different across the two peel types. Asp, Glu, and Pro had the highest levels but Cys, Met, and Tyr had the lowest levels in mandarin peel. Some mineral nutrients also had significant changes in RD peel. Analyses revealed that higher N, Mg and Cu levels occurred in mature peel, while other mineral nutrients showed no differences (Table 3). To further characterize the mineral nutrient changes in RD peel, four candidate mineral nutrients were determined during fruit development. Analyses over the developmental course suggested that Mg content was constantly higher in RD peel relative to CK (Figure 3).

**Table 1:** Classification of metabolites with significant difference between CK and RD peels.

**Classification**	**Putative Name**	**Fold change (PD/CK ratio)**	***t*-test**
Sugars	1-Kestose	0.54	0.036
	1,6-anhydro- beta-Glucose	0.57	0.022
	Raffinose	0.65	0.043
	Galactose	1.54	0.015
	Psicose	2.02	0.036
	Sorbose	2.19	0.003
	Galactonic acid	0.77	0.049
Organic acids	Glyoxylic acid	0.57	0.020
	Benzoic acid	0.58	0.045
	Shikimic acid	0.60	0.002
	methyl-Malonic acid	0.64	0.004
	1-Pyrroline-3-hydroxy-5-carboxylic-acid	1.62	0.001
	2-oxo-Gulonic acid	2.59	0.013
	Lactic acid	4.06	<0.0001
	Glyceric acid	4.54	0.004
Amino acids and derivatives	Phenylalanine	0.52	0.036
	3-cyano-Alanine	1.71	0.021
	Threonine	1.83	0.019
	Glycine	2.16	<0.0001
	Norvaline	2.60	0.000
	Citrulline	4.80	0.000
	beta-Homoserine	5.51	<0.0001
	Glutamine	8.43	0.01
	Indole-3-lactic acid	3.21	0.004
Alcohols	Viburnitol	0.69	0.005
Heterocyclic compound	Similar to Lumichrome	5.91	<0.0001
Amines	Butyro-1,4-lactam	1.31	0.027
	Propylamine-2,3-diol	1.56	0.019
Others	2-hydroxy-Pyridine	0.62	0.042
	Epinephrine	1.72	0.031

*The relative concentrations of metabolites are based on peak areas, and five individually biological replicates were conducted for a t-test. The RD/CK ratio was calculated on the mean values*.

**Table 2:** Amino acids in CK and RD peels. Values are mean ± *SD* of three biological replicates.

	**CK (mg/100g DW)**	**PD (mg/100g DW)**
Asp	0.69 ± 0.02	0.78 ± 0.02^**^
Thr	0.15 ± 0.005	0.18 ± 0.003^**^
Ser	0.19 ± 0.02	0.22 ± 0.02^**^
Glu	0.48 ± 0.02	0.56 ± 0.01^**^
Pro	0.52 ± 0.02	0.49 ± 0.01^*^
Gly	0.20 ± 0.006	0.23 ± 0.004^**^
Ala	0.27 ± 0.02	0.30 ± 0.005^**^
Cys	0.07 ± 0.02	0.04 ± 0.003^**^
Val	0.19 ± 0.009	0.20 ± 0.004^**^
Met	0.03 ± 0.008	0.02 ± 0.004^*^
Ile	0.13 ± 0.004	0.14 ± 0.0103^**^
Leu	0.25 ± 0.005	0.28 ± 0.005^**^
Tyr	0.02 ± 0.03	0.07 ± 0.005^**^
Phe	0.18 ± 0.01	0.23 ± 0.005^**^
Lys	0.21 ± 0.02	0.27 ± 0.02^**^
His	0.09 ± 0.003	0.12 ± 0.004^**^
Arg	0.20 ± 0.003	0.21 ± 0.10N

*Asterisk and double asterisk mean significant difference at P < 0.05 and P < 0.01, respectively. N means no significant difference*.

**Table 3:** Mineral nutrients in mature CK and RD peels.

	**N (%)**	**P (%)**	**K (%)**	**Ca (%)**	**Mg (%)**	**Zn (ug/g)**
CK	0.79 ± 0.02	0.04 ± 0.01	1.13 ± 0.04	0.50 ± 0.02	0.07 ± 0.01	4.40 ± 0.23
RD	0.88 ± 0.03^*^	0.05 ± 0.001	1.00 ± 0.11	0.51 ± 0.05	0.19 ± 0.01^*^	4.81 ± 0.06
	**Cu (ug/g)**	**Mn (ug/g)**	**Fe (ug/g)**		**B (ug/g)**	**Mo (ug/g)**
CK	1.87 ± 0.13	7.58 ± 2.09	28.99 ± 12.23		17.91 ± 0.13	0.04 ± 0.00
RD	2.96 ± 0.44^*^	9.71 ± 0.45	29.15 ± 9.59		18.60 ± 0.63	0.05 ± 0.02

*Values are mean ± SD of three biological replicates. Asterisk means significant difference at P < 0.05*.

### Changes in global gene expression responding to peel RD

During fruit development, CK peel at 30 DAFB, RD peel at 30 DAFB, CK peel at 80 DAFB, RD peel at 80 DAFB, CK peel at 170 DAFB, and RD peel at 170 DAFB were chosen to construct sequencing libraries, named CK30, RD30, CK80, RD80, CK170, and RD170, respectively. After filtering, more than 65.5 million clean sequence reads for each library were generated, for a total of 39.83 GB of sequence data (Table S2). Of the total clean reads from the samples, 70.5–72.2% were unique matches with the sweet orange (*Citrus sinensis*) genome (http://citrus.hzau.edu.cn/orange/) while multi-position match reads were 4.0–5.1%. Finally, 20,339 expressed genes in CK30, 20,997 expressed genes in RD30, 20,659 expressed genes in CK80, 20,711 expressed genes in RD80, 20,554 expressed genes in CK170 and 20,501 expressed genes in RD170 were detected based on the reference genome. Gene-expression levels were determined using the fragments per kilobase of transcript per million mapped reads (FPKM) method. A total of 4,855, 1,164, and 2,526 DEGs were detected at 30, 80, and 170 DAFB, respectively (Table S3). Of these, two peels at 30 DAFB exhibited the most DEGs with 3,863 (79.6%) upregulated and 992 (20.4%) down-regulated. The differentially expressed transcription factors between CK and RD peels were also analyzed. A total of 43 differentially expressed transcription factor families were found during RD development. Of these, 42 families were expressed differently at 30 DAFB, with 241 up-regulated and 55 down-regulated transcription factors. MYB and bHLH families predominated, with 40 and 32 transcription factors differentially expressed, respectively (Table S4).

**Figure 4:**
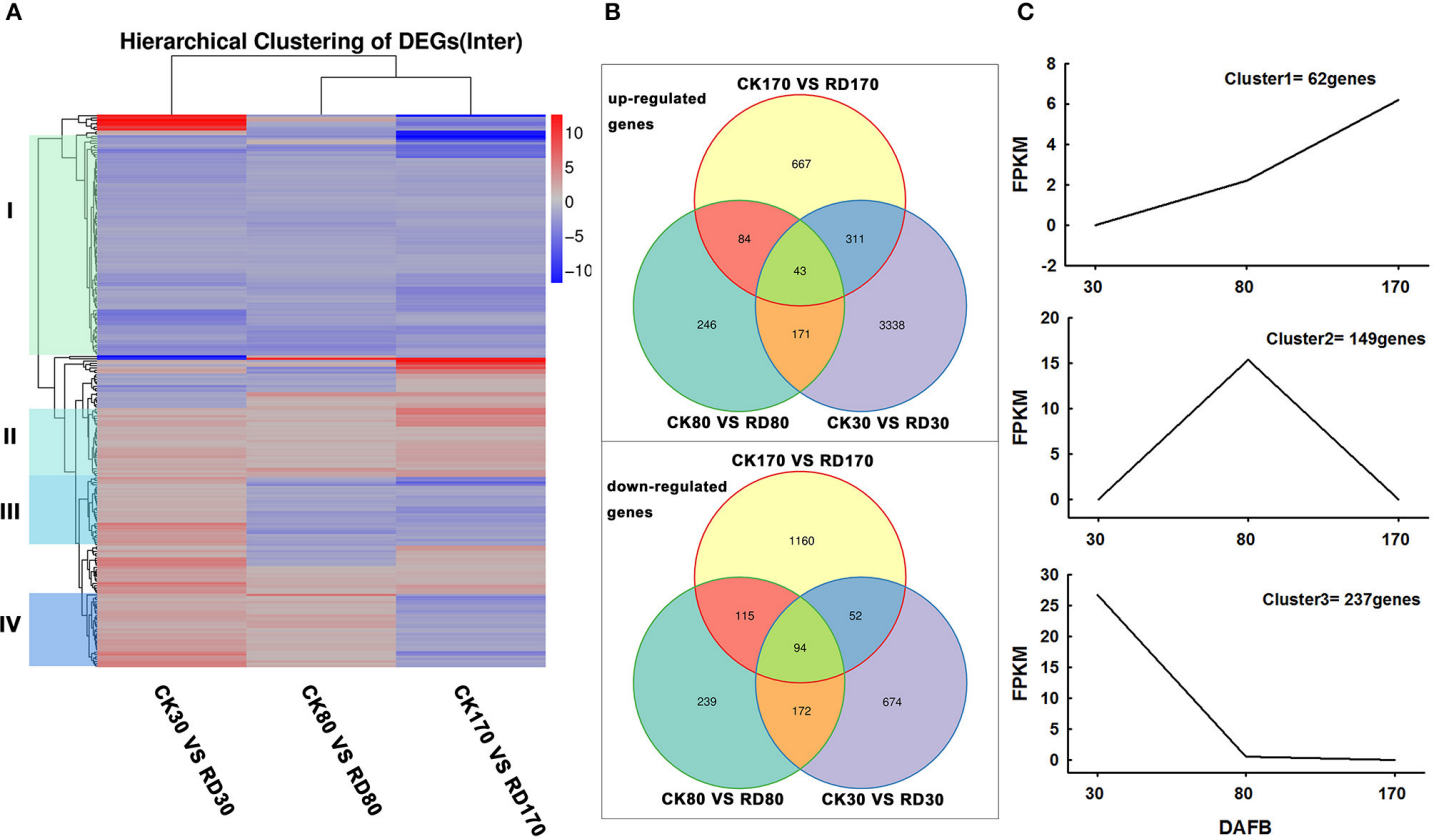
Expression patterns of differentially expressed genes (DEGs). Hierarchical clustering **(A)** and Venn diagrams **(B)** of DEGs at three development stages. Clustering analyses of developmentally altered genes **(C)**.

**Figure 5:**
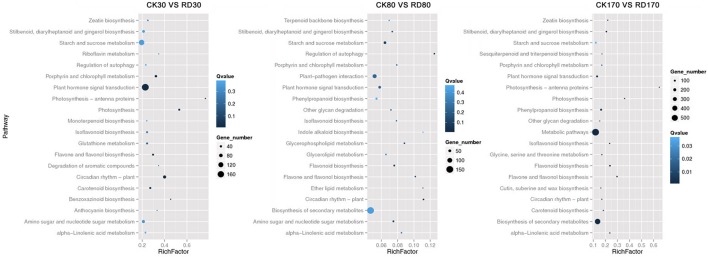
Overview of top 20 altered pathways in RD peel at three development stages.

Hierarchical cluster analyses was performed using the log-[FPKM (RD/CK)] values of samples at three stages. Results showed that during the three stages, the DEGs in peel were divided into more than five groups (Figure 4A). Of these groups, four were predominant: in group I, genes downregulated at all three development stages; in group II, genes upregulated at all three development stages; in group III, genes upregulated at cell division stage and downregulated at cell enlargement and maturity stage and in group IV, genes upregulated at cell division and enlargement stages and then downregulated at maturity stage. This supported the notion that different genes worked during fruit puffing development. To analyse the metabolic and regulatory pathways involved in peel RD, a comparison of DEGs at three development stages was made. In RD peel, 3,863 genes at 30 DAFB, 544 genes at 80 DAFB and 1,105 genes at 170 DAFB were up-regulated relative to CK peel, with three groups sharing 43 genes (Figure 4B up). In contrast, 992 genes at 30 DAFB, 620 genes at 80 DAFB and 1,421 genes at 170 DAFB were down-regulated, with 94 genes common to the three groups (Figure 4B down). To identify DEGs that showed alternation in their expression during peel development and common or different pathways that altered developmentally, they were clustered according to their expression patterns. Three patterned DEGs with up-up-regulation, up-down-regulation and down-down-regulation during peel development were identified. In these three patterns, down-down-regulation had the most genes (237) while up-up-regulation contained the least genes (63) (Figure 4C). All DEGs were assigned to metabolism pathways (Table S5). At 30 DAFB, plant hormone signal transduction with 173 DEGs and starch and sucrose metabolism with 130 DEGs were altered most significantly in RD peels. Subsequently, pathways involved in plant circadian rhythms, amino sugar, and nucleotide sugar metabolism and stilbenoid, diarylheptanoid, and gingerol biosynthesis were also affected. At 80 and 170 DAFB, the metabolic pathway and biosynthesis of secondary metabolites possessed the most DEGs that accorded with fruit development and carotenoid biosynthesis (Figure 5).

### Starch, sugar, and acid accumulation in RD peel of satsuma mandarin fruit

**Figure 6:**
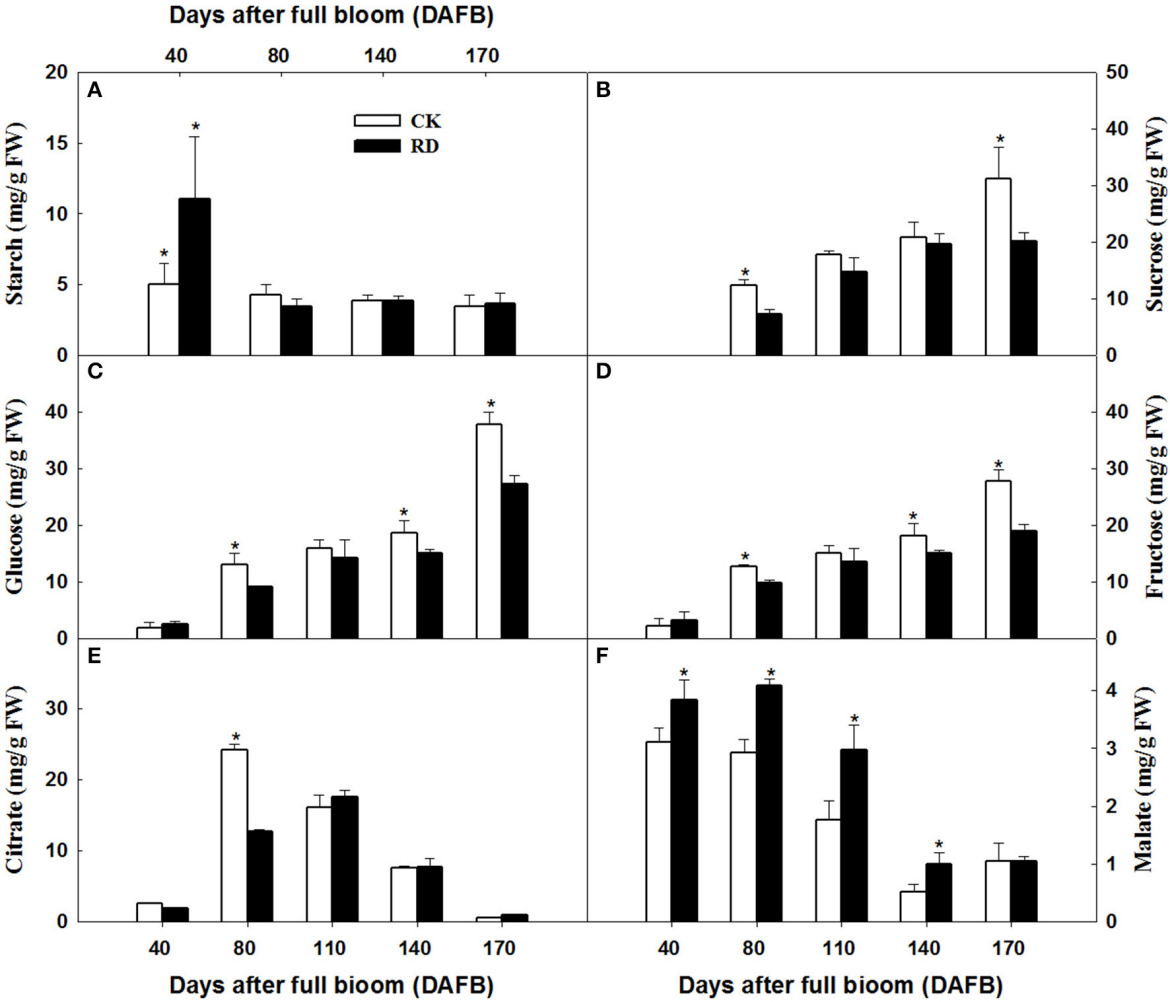
Starch **(A)**, sucrose **(B)**, glucose **(C)**, fructose **(D)**, citrate **(E)**, and malate **(F)** accumulation in CK and RD peel during fruit development. Asterisk means significant difference at *P* < 0.05.

**Figure 7:**
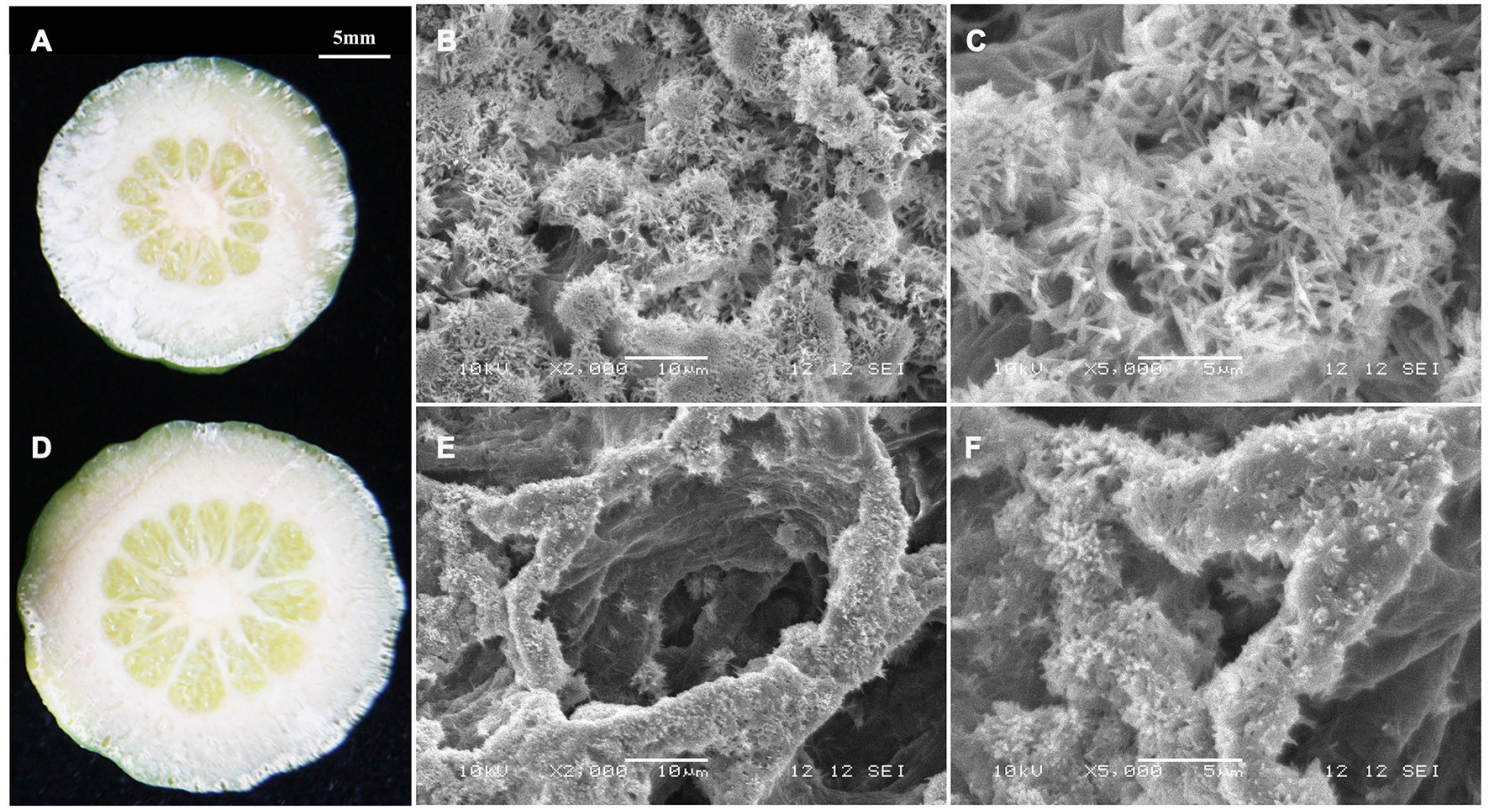
Morphology of starch in CK peel **(A–C)** and RD peel **(D–F)** at 30 DAFB.

Transcriptome analyses showed that gene expression in starch and sucrose pathways alternated significantly at different stages, indicating the important roles of the pathways in RD peel formation. Therefore, starch and sucrose contents in the peel during fruit development were measured. The results showed that starch was up to 10 mg/gFW in RD peel at 30 DAFB, significantly higher than 5 mg/gFW in CK peel. Subsequently, starch content in both peels decreased quickly and remained at 6–9% until fruit maturity (Figure 6A). Starch difference between the two peels was evident in content as well as in morphology. Due to the abundant starch in citrus fruitlet, starch overflow after cutting could be observed easily. *In situ* observation under SEM, starch in CK peel had longer chains than starch in RD peel, and starch accumulation was much denser in RD peel than in CK peel (Figure 7).

During peel development, two or three sampling dates out of five indicated significantly higher sucrose, glucose and fructose levels in CK peel than in RD peel. Three sugars all had lower levels in RD peel at 170 DAFB (Figures 6B–D). Between the two peels, there was no difference in citrate at most sampling dates. Malate was higher in RD peel than in CK peel between 40 and 140 DAFB, but the difference disappeared at 170 DAFB (Figures 6E,F). Because peel is the media tissue for leaf photosynthate transporting to fruit pulp, there should be links in sugars and acids between peel and pulp. Analyses showed that pulp of RD fruit had lower sucrose, glucose, and fructose content at 170 DAFB but showed no difference in organic acids, similar to patterns in RD peel (Figure S1).

### Hormone signal transduction in RD peel of satsuma mandarin fruit

**Figure 8:**
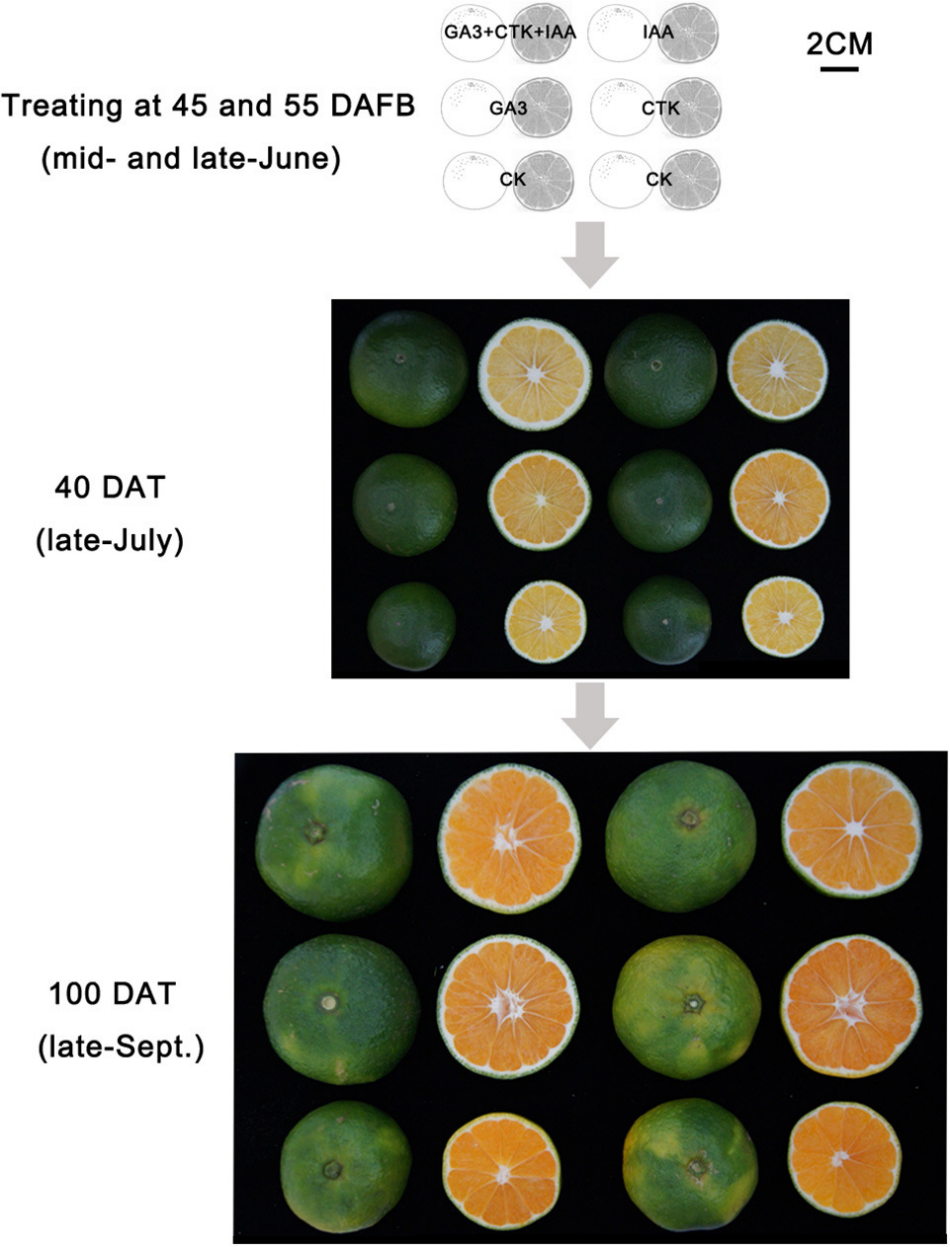
Fruit development after hormone treatment.

Of the pathways revealed by RNA-sequencing, plant hormone signal transduction was clearly and significantly affected in RD peel, especially at 30 DAFB. Based on the observations and previous reports, effects of auxin, cytokinin, and gibberellin, which are likely associated with peel RD, were further studied. Exogenous applications of auxin (IAA), gibberellin (GA_3_), cytokinin (CTK) or a mixture (IAA+GA_3_+CTK), were used to soak the normal fruit to identify their roles in RD. After twice soaking at 40 and 50 DAFB, fruits of all treatments enlarged significantly at 85 and 115 DAFB compared to CK, while mixture treatment drove the largest fruit enlargement at 85 DAFB. However, there was no fruit size difference between IAA, GA_3_, CTK, and mixture treatments except a slightly larger fruit in mixture treatment at 85 DAFB. Along with fruit enlargement, fruit peel thickness changed significantly depending on hormones (Table 4). Compared to CK, thickened peel was found at 85 DAFB, a month after treating, and peel thickness was 3.56, 3.27, and 3.78 mm in IAA, GA_3_, and mixture treatments, respectively. CTK and CK treatments were thinner, <2.9 mm. Subsequently at 115 DAFB, with RD appearing, the peel thickness in IAA, CTK, and mixture treating fruit showed a slight decrease, but in GA_3_ treated fruit the thickness increased, contributing to peel RD (Figure 8, Table 4). In the bigger fruit after hormone treatment, larger steles at 85 DAFB and hollow steles at 115 DAFB were observed (Figure 8).

**Table 4:** Changes of fruit size and peel thickness after hormone treatment.

	**DAFB**	**CK**	**IAA**	**CTK**	**GA3**	**IAA+CTK+GA3**
Fruit diameter (mm)	85	42.80 ± 1.47c	51.20 ± 3.25b	50.27 ± 3.12b	51.13 ± 3.86b	53.35 ± 2.73a
	115	60.20 ± 2.90b	67.50 ± 5.55a	67.43 ± 11.60a	68.45 ± 5.94a	68.30 ± 5.11a
Peel thickness (mm)	85	2.41 ± 0.42d	3.56 ± 0.36ab	2.89 ± 0.39c	3.27 ± 0.43b	3.78 ± 0.55a
	115	2.29 ± 0.48d	2.49 ± 0.68cd	2.87 ± 0.21bc	3.38 ± 0.43a	3.07 ± 0.75ab

*Values are mean ± SD of thirty individual replicates. Different letters mean significant difference at P < 0.05*.

**Figure 9:**
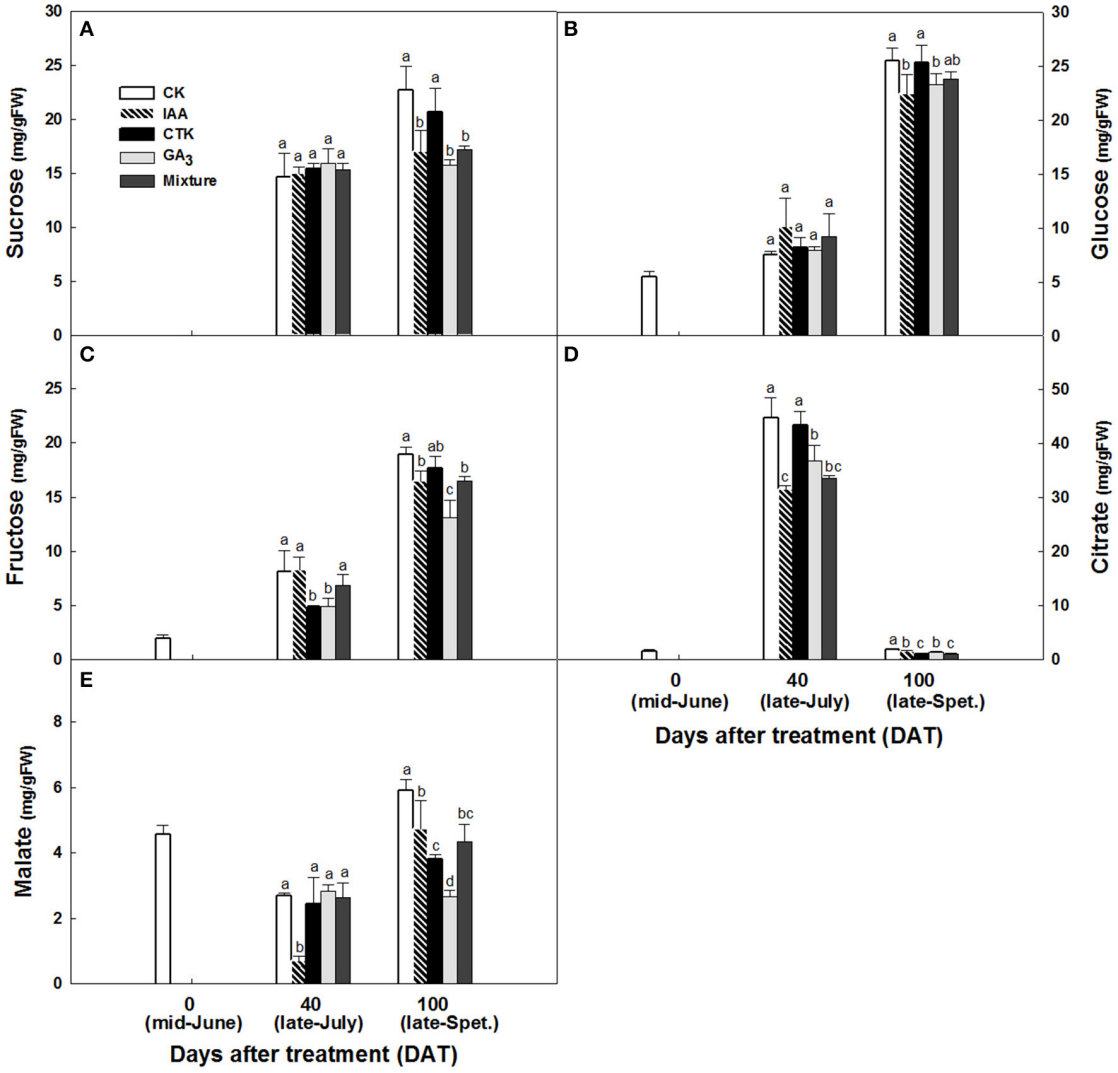
Sucrose **(A)**, glucose **(B)**, fructose **(C)**, citrate **(D)** and malate **(E)** accumulation in peel after hormone treatment. Different letters mean significant difference at *P* < 0.05.

Accumulation of sugars and organic acids in fruit were altered after hormone treatment. From June to September, sugars in peel accumulated constantly and significantly whether treated by hormones or not, with an exception of GA_3_ on sucrose (Figures 9A–C). Peel sucrose and glucose contents showed no difference between hormone treatments at 40 days after treatment (DAT) while these in IAA, GA_3_, and mixture treatments were lower than that in CK and CTK treatment at 100 DAT (Figures 9A,B). Peel fructose was lower stably in GA_3_ treatment since 40DAT (Figure 9C). Citrate increased significantly from 0 to 40 DAT and then decreased sharply in all peels, while malate decreased slightly from 0 to 40 DAT and increased after that. Peel citrate decreased after all hormone treatments except CTK at 40 DAT. In peel at 100 DAT, CTK, and mixture induced the lowest citrate (Figure 9D). Malate in peel declined after hormone treatments and that occurred 100 DAT mainly (Figure 9E). In pulp, total soluble solids (TSS) also decreased after IAA, GA_3_, and mixture treatments at 100 DAT (Figure S2A). Sucrose showed slight decrease in IAA and mixture treatments at 100 DAT (Figure S2B). Consistent with the TSS pattern, fructose and glucose were also lower in IAA, GA_3_, and mixture treatments than in CK and CTK treatments (Figures S2C,D). Pulp in CTK treated fruit exhibited lower citrate levels than CK and other hormone treated fruit from 40 to 100 DAT. Malate in the pulp of IAA, GA_3_, and CTK treated fruit was higher than in CK and mixture treated fruit at 100 DAT (Figures S2E,F).

### Genes initially responsible for peel RD

The genes potentially associated with initial RD were assessed in this study. Because gibberellin works on both tissue growth and starch metabolism, genes involved in gibberellin signal transduction at 30 DAFB were most probably associated with peel RD. Of 4,857 DEGs at 30 DAFB, 243 DEGs were assigned to the plant hormone signal transduction pathway. Among these, 49 genes belonged to gibberellin signal transduction, and contained 19 GA insensitive dwarf (GID) genes, 14 DELLA genes and 16 transcription factors. In the gibberellin signal transduction pathway, *GID* and transcription factors positively regulate tissue growth, but DELLA negatively regulates it. As such, 15 out of 49 genes were further screened. The expression levels of 13 genes were higher in RD30 than in CK30, but two DELLA genes, *DELA7* and *DELA8*, had downregulated expression in RD30 (Table 5). To confirm this gene expression pattern in the gibberellin signal transduction pathway, 21 DEGs containing 15 potentially crucial genes and 6 *bHLH* transcription factors were selected for expression profile analyses using quantitative RT-PCR. The results showed similar expression patterns to RNA-sequencing results, despite some quantitative differences (Figure S3).

**Table 5:** List of important DEGs initially responding to peel roughing disorder.

**Gene name**	**Transcript ID**	**Log**_**2**_ **(FPKMratio)**			**RD FPKM**		
		**RD30/CK30**	**RD80/CK80**	**RD170/CK170**	**30 DAFB**	**80 DAFB**	**170 DAFB**
*GID1e1*	Cs5g34000	4.13	–	–	4.73	1.83	0.19
*GID1b1*	Cs8g05610	3.46	–	–	15	10.15	2.6
*GID1b2*	Cs7g05330	2.92	–	–	5.59	4.61	3.66
*GID1*	Cs8g05590	2.85	–	–	6.47	3.2	0.65
*GID1e2*	Cs5g34010	2.38	–	–	21.71	16.26	3.34
*GID1c*	Cs5g19100	1.83	–	–	18.54	11.1	7.62
*GID2*	Cs3g23040	1.03	–	–	60.99	60.06	40.68
*DELLA7*	Cs2g01990	−1.74	–	–	150.59	116.09	148.67
*DELLA8*	Cs4g12130	−2.20	−1.68	−1.14	88.12	59.84	35.24
*bHLH51*	Cs7g30860	4.20	–	–	6.63	3.37	1.76
*bHLH 30*	Cs3g17300	3.34	–	–	5.78	4.21	7.9
*bHLH 122*	orange1.1t03173	2.06	1.12	–	11.44	10.61	4.9
*bHLH 56*	Cs3g23320	1.97	–	–	3.37	1.8	0.61
*bHLH 7*	Cs9g13980	1.53	–	–	22.69	17.04	18.46
*bHLH 122.1*	Cs7g02010	1.08	−1.10	–	33.59	15.24	11.38

## Discussion

RD is a physiological fruit disorder in Satsuma mandarins, occurring in most production areas. Although Satsuma mandarin RD has been described in previous studies, it has not been comprehensively examined and interpreted. In the present study, the exterior and interior characteristics of RD fruit were observed, with particular emphasis on peel RD. Unlike puffing disorder, which is characterized by a split between peel and pulp and the production of aerial spaces through dissolution of albedo (Kuraoka, 1962; Martinelli et al., 2015), RD is mainly characterized by a rough fruit surface, enlarged fruit size and thicker peel. Apart from the rough appearance, it also exhibits other quality barriers such as delayed degreening, enlarged segments, thickened flavedo and albedo, and lignified segment membranes and juice sacs (Figures 1A,B). RD fruit follows an altered development pattern involved in both fruit size and peel. It appears that the imbalance of source/sink ratio originating from some physiological factors causes the constant and vigorous growth pattern of RD fruit (Figures 1C,D).

At fruit maturity stage, more than 850 metabolites were found in CK and RD peels. Most differences (and the largest differences) were in the intermediates of primary metabolism involved in sugar, organic acid, and amino acid metabolism (Table 1). In particular, both targeted and untargeted measurements revealed significantly higher amino acid contents in RD peel, suggesting upregulated amino acid metabolism (Table 2). Meanwhile, the higher Glu content might contribute to the chlorophyll precursor, which is associated with delayed degreening of RD peel. Magnesium and nitrogen play important roles in citrus chlorophyll accumulation (Yin et al., 1998; Huang et al., 2014). Mineral nutrient analyses showed that RD peel contained more magnesium and nitrogen than CK peel throughout fruit development, which could be responsible for the delayed degreening (Figure 3, Table 3). The month after full bloom is a key period for RD development. Following the three developmental stages for Satsuma mandarins (Kubo and Hiratsuka, 1998), cell division occurred before 60 DAFB in this work when the cell amount and thickness of the peel changed. Upward fruit of “Okitsu wase” mandarin exhibited significant RD at the end of cell division stage (Kubo and Hiratsuka, 1998). In “Guoqing No. 1” mandarins, where defruiting induces RD, histological analyses showed that loose cell arrangement in albedo occurred after 21 DAFB and rough cell arrangement in flavedo appeared after 28 DAFB (Liu, 2012). In this study, SEM analyses showed that increased cell layers, enlarged cell volume and looser cell arrangements in RD peel were present at 30 DAFB, and these differences remained during subsequent development stages (Figure 2). All the above results indicate that vigorous cell division supplied a basis for disorder development at subsequent stages and was an early symptom of peel RD, which generally happened within a month after full bloom.

Citrus fruit set needs sufficient carbohydrate support (Mehouachi et al., 2000). In Satsuma mandarin, source–sink imbalance assays through defoliation and sucrose stem injection demonstrated that fruit set and fruitlet growth are highly dependent on carbohydrate availability (Iglesias et al., 2003, 2006). The major carbohydrates in citrus fruit are starch at early development stage and sugars and acids at middle and late development stages. Branch girdling on “Okitsu wase” mandarins performed at anthesis temporarily delayed the initial process of natural fruitlet drop, while higher carbohydrates (hexose and starch) and GAs contributed to the delay of fruitlet abscission (Mahouachi et al., 2009). In this work, defruiting at fruit set caused significantly more starch accumulation in RD peel, a similar effect to branch girdling at anthesis. These results indicated that the strategies enhancing carbohydrate accumulation in fruitlets were beneficial to fruit set and fruitlet growth. Furthermore, the starch chain was affected in RD peel in addition to an increase in starch content (Figures 6A, 7). This is probably associated with gibberellin signal activity in RD peel, which is an important accelerator for starch hydrolysis. Gibberellin-mediated starch hydrolysis had been identified in avocados (Leshem et al., 1973), maize (Cao and Shannon, 1997), *Medicago sativa* L. and other crops (Kepczynska and Zielinska, 2006). Thus, increased gibberellin signal transduction likely promotes fruitlet development and drives starch hydrolysis in RD peel.

As the other major carbohydrates in Satsuma mandarins, sugars and acids determine fruit flavor. In Satsuma mandarin pulp, sugars increase from early September until fruit maturity, and acids increase until mid-August and then decrease until fruit maturity (Zhao, 2008). In the juice of Satsuma mandarin fruit with RD, total sugar and its components were all lower in RD fruit than CK fruit from mid-August to early December. Total acid and citrate levels were higher in RD fruit than CK fruit from early August to mid-September but malate displayed the reverse pattern. However, all the acid differences between CK and RD fruit disappeared after late September (Kubo and Hiratsuka, 1998). Low fruit load, possibly inducing RD, led to lower sucrose in Satsuma mandarin pulp (Kubo et al., 2001). Results in this study indicated that there is less sugar in RD peel and pulp, especially at fruit maturity stage (Figure 6 and Figure S1); IAA, CTK, GA_3_ and mixture treatments could lead to lower sugars and acids in both peel and pulp, with GA_3_ working efficiently and stably (Figure 9 and Figure S2). Together, these results suggest that RD negatively affects sugar and acid accumulation in Satsuma mandarin fruit through GA_3_ playing an important role.

**Figure 10:**
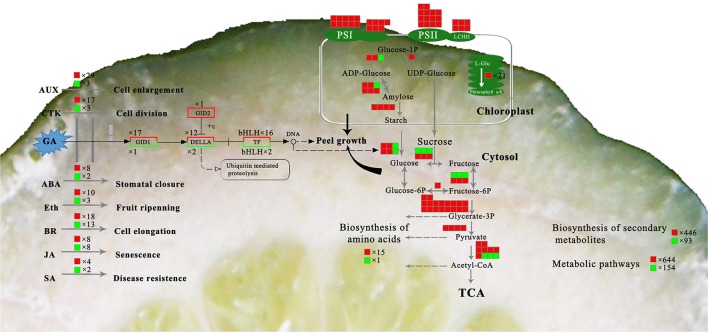
Overview of the major metabolic pathways involved in peel RD. The box background in red indicates up-regulated genes while green indicates down-regulated genes.

It is generally accepted that GA operates in fruit set in citrus (Talon, 1992; Ben-Cheikh et al., 1997; Mahouachi et al., 2009). GA is an activator of cell division and cell enlargement processes, and its presence is generally associated with the initiation of both phases. In Tankan (*Citrus tankan Hayata*), exogenous GA_3_ induced RD due to increased flavedo thickness and a prolonged cell division stage (Liu et al., 1988). A similar effect of exogenous GA_3_ was also found in “Okitsu wase” mandarin, but IAA, BA and ABA had no effect on RD (Kubo and Hiratsuka, 2000). In addition, application at early development stage also means that GA results in RD. GA_3_ application in late June led to peel RD but did not do so in mid-July. GA content was higher in RD peel in late June but the difference disappeared in mid-July (Kubo and Hiratsuka, 2000). Similarly, the effect of GA on biological function depending on application time was also found in citrus flowering induction (Guardiola et al., 1982; Lord and Eckard, 1987; Goldberg-Moeller et al., 2013). Together with our tests of GA_3_, IAA, and CTK on RD, these findings indicate that GA plays a crucial role in peel RD, and the early development stage is a key period for GA-induced RD in Satsuma mandarin. In the gibberellin signal transduction pathway, expression of *CsGIDs, CsDELAs*, and *bHLH* families were affected at 30 DAFB in response to peel RD. Of these genes, transcription factors were involved in both peel growth and starch hydrolysis in peel, indicating their crucial roles in initiating RD (Figure 10). In fruit, the *bHLH* family is associated with peel pigment metabolism. The *bHLH* family regulates anthocyanin metabolism in apples (An et al., 2012; Xie et al., 2012; Meng et al., 2016), Chinese bayberry (Liu et al., 2013), and blood oranges (Sun et al., 2014). The *bHLH* family is also involved in fruit development in model plants (Groszmann et al., 2008, 2011). In Satsuma mandarins, *CubHLH1* modulates carotenoid metabolism in the peel, which can be accelerated or slowed by ethylene or gibberellin, respectively (Fujii et al., 2007, 2008; Endo et al., 2016). In this study, expression of all six *bHLHs* involved in gibberellin transduction (*bHLH51, bHLH 30, bHLH 122, bHLH 56, bHLH 7*, and *bHLH 122.1*) were upregulated, indicating their roles in peel RD development.

## Conclusion

Satsuma mandarin fruit RD could be initiated at the month after full bloom. Metabolome analysis of RD peel showed that content of Mg and many carbohydrates were significantly affected. RNA-sequencing suggested there were many more DEGs responding to RD at early fruit development stage than at subsequent stages. This study revealed starch metabolism and GA signal transduction pathways were changed significantly. Several aspects might account for peel RD, including: (a) The RD peel had more vigorous carbohydrate metabolism at early fruit development stage relative to CK peel; (b) GA played a crucial role in RD peel initiation through cell division, peel growth and carbohydrate metabolism; (c) The higher Mg content might contribute to the chlorophyll biosynthesis, carbohydrate accumulation and delayed degreening in RD peel.

## Data access

RNA-sequencing data are submitted to Gene Expression Omnibus (GEO) repository under accession No. GSE100512 (http://www.ncbi.nlm.nih.gov/geo/query/acc.cgi?acc=GSE100512).

## Author contributions

X-PL, S-YC, and S-XX conceived and designed research. X-PL, JX, and F-FL conducted experiments. Z-MZ and X-CM contributed new reagents and analytical tools. X-PL and F-FL analyzed data. X-PL wrote the manuscript and S-XX revised the manuscript. All authors read and approved the manuscript.

### Conflict of interest statement

The authors declare that the research was conducted in the absence of any commercial or financial relationships that could be construed as a potential conflict of interest.

## Supplementary material

Figure S1 Sucrose **(A)**, fructose **(B)**, glucose **(C)**, citrate **(D)**, and malate **(E)** accumulation in CK and RD pulp during fruit development. Asterisk means significant difference at *P* < 0.05.

Figure S2 TSS **(A)**, sucrose **(B)**, fructose **(C)**, glucose **(D)**, citrate **(E)**, and malate **(F)** accumulation in pulp after hormone treatments. Different letters mean significant difference at *P* < 0.05.

Figure S3 Relative expression of genes involved in GA signal transduction. Values are mean ± *SD* of three biological replicates calibrated against the amount of β-actin control expression. Numbers at the top of the columns represent the FPKM values calculated from sequencing data.
