# Supplementary figures and images for: Transcriptome and Metabolome Analyses Provide Insights into the Occurrence of Peel Roughing Disorder on Satsuma Mandarin (Citrus unshiu Marc.) Fruit
Source: Front Plant Sci. 2017 Nov 7;8:1907. doi: 10.3389/fpls.2017.01907 (PMC5682035; doi:10.3389/fpls.2017.01907)

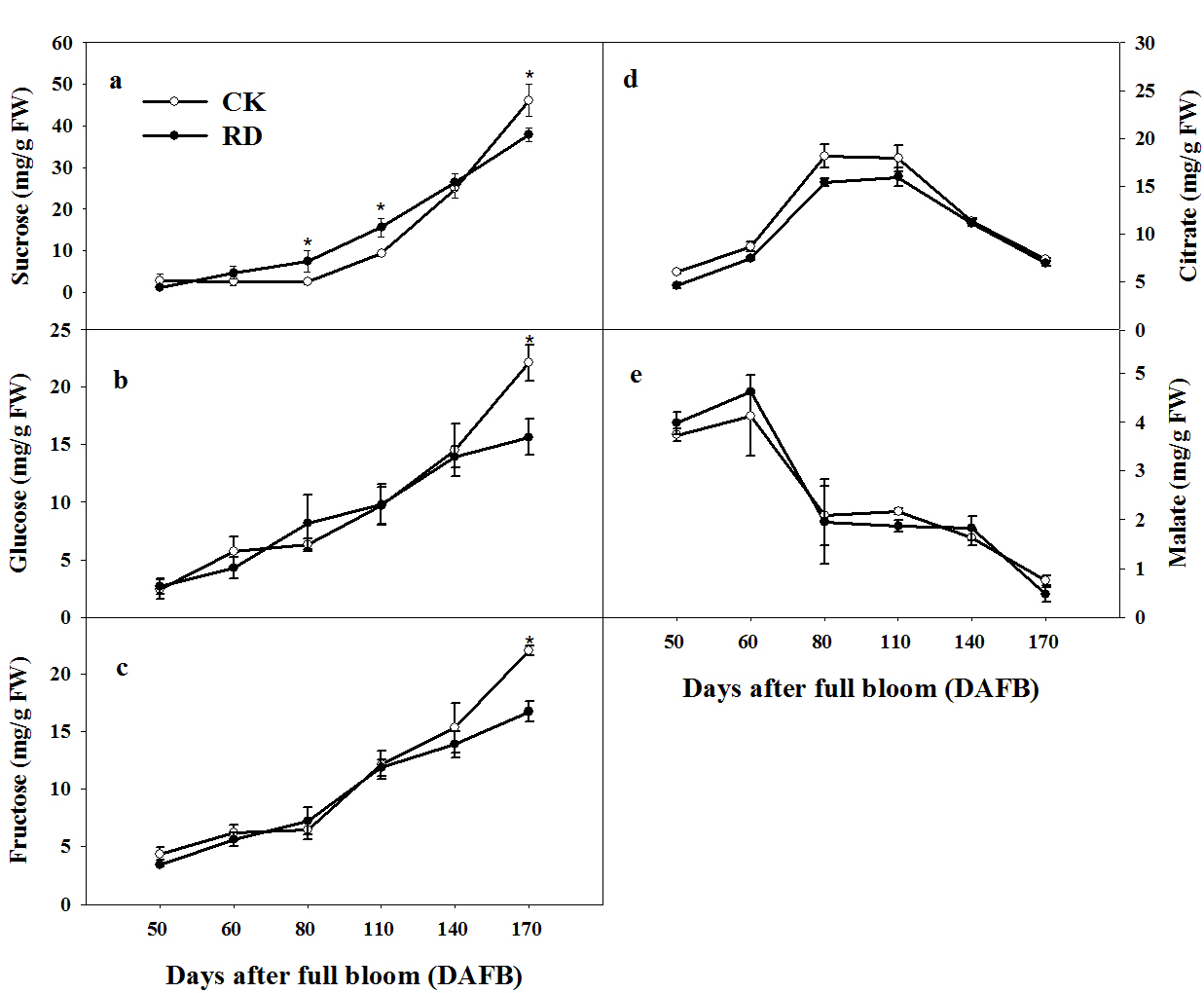

Supplement: Figure S1 — Sucrose (A), fructose (B), glucose (C), citrate (D), and malate (E) accumulation in CK and RD pulp during fruit development. Asterisk means significant difference at P < 0.05. [file Image1.JPEG]

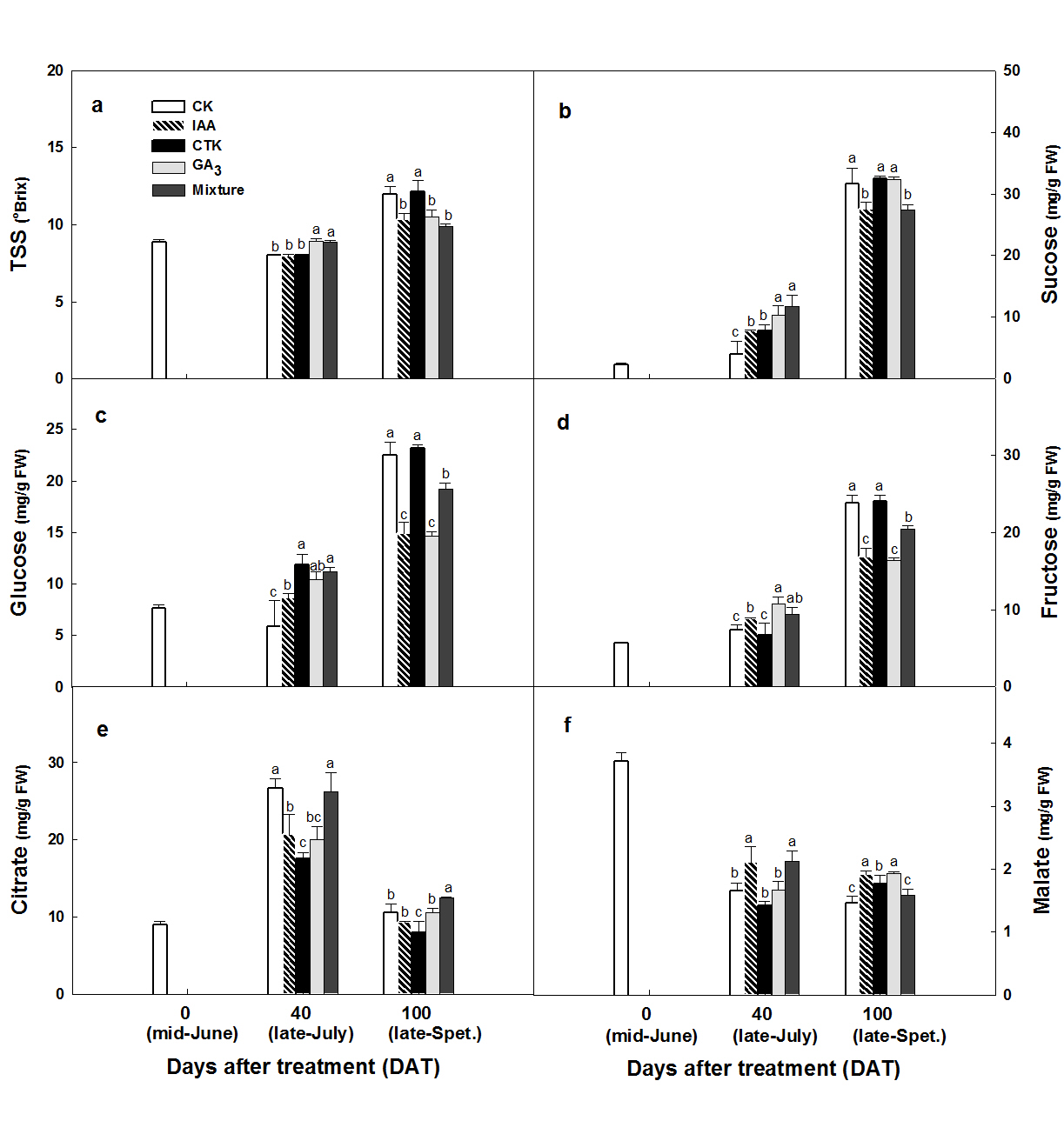

Supplement: Figure S2 — TSS (A), sucrose (B), fructose (C), glucose (D), citrate (E), and malate (F) accumulation in pulp after hormone treatments. Different letters mean significant difference at P < 0.05. [file Image2.JPEG]

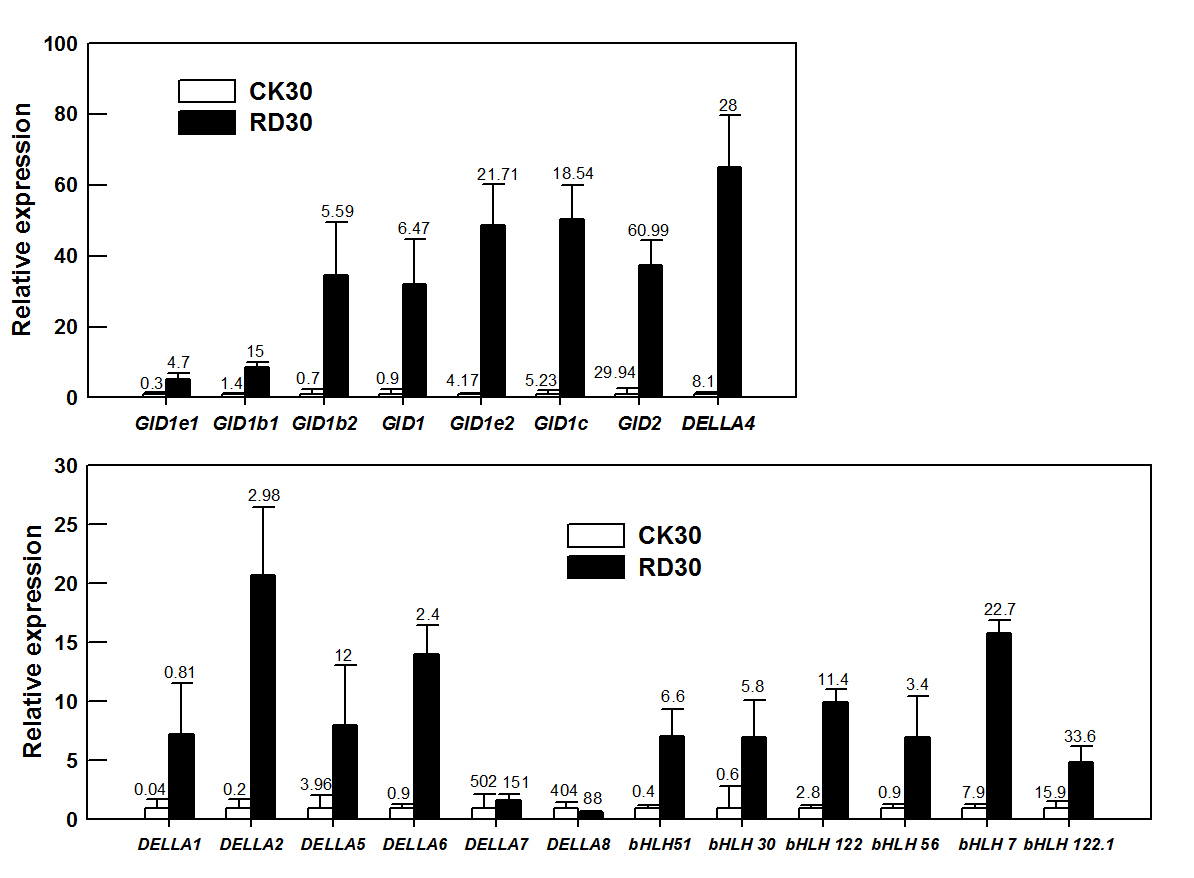

Supplement: Figure S3 — Relative expression of genes involved in GA signal transduction. Values are mean ± SD of three biological replicates calibrated against the amount of β-actin control expression. Numbers at the top of the columns represent the FPKM values calculated from sequencing data. [file Image3.JPEG]
