# Supplementary material for: Transcriptome and Metabolome Analyses Provide Insights into the Occurrence of Peel Roughing Disorder on Satsuma Mandarin (Citrus unshiu Marc.) Fruit
Source: Front Plant Sci. 2017 Nov 7;8:1907. doi: 10.3389/fpls.2017.01907 (PMC5682035; doi:10.3389/fpls.2017.01907)
Supplement: Supplementary file 5 [file Table2.DOCX]

**Table S2 Summary statistics of the transcriptome reads.**

|  | **CK30** | **RD30** | **CK80** | **RD80** | **CK170** | **RD170** |
| --- | --- | --- | --- | --- | --- | --- |
| Total Reads | 66,890,116 | 66,555,840 | 66,678,438 | 66,798,008 | 65,844,114 | 65,505,894 |
| Total Base Pairs | 6.69Gb | 6.66Gb | 6.67Gb | 6.68Gb | 6.58Gb | 6.55Gb |
| Expressed genes | 20339 | 20997 | 20659 | 20711 | 20554 | 20501 |
| Total Mapped Reads | 51,771,903(77.40%) | 51,063,475(76.72%) | 50,279,803(75.41%) | 50,490,181(75.59%) | 4,982,3585(75.67%) | 49,379,174(75.38%) |
| Unique Match | 48,557,715(72.59%) | 48,078,967(72.24%) | 47,102,294(70.64%) | 47,075,322(70.47%) | 4,7174,719(71.65%) | 46,636,254(71.19%) |
| Multi-position Match | 3,214,188(4.81%) | 2,984,508(4.48%) | 3,177,509(4.77%) | 3,414,859(5.11%) | 2,648,866(4.02%) | 2,742,920(4.19%) |
