# Supplementary material for: Transcriptome and Metabolome Analyses Provide Insights into the Occurrence of Peel Roughing Disorder on Satsuma Mandarin (Citrus unshiu Marc.) Fruit
Source: Front Plant Sci. 2017 Nov 7;8:1907. doi: 10.3389/fpls.2017.01907 (PMC5682035; doi:10.3389/fpls.2017.01907)
Supplement: Supplementary file 6 [file Table3.DOCX]

**Table S3**  **Differentially expressed genes across libraries.** All the genes mapped to the reference sequence were examined for their expression differences across the four libraries. Numbers of differentially expressed genes represent across sense transcripts, using the threshold with a FDR (False Discovery Rate) ≤0.001 and the absolute value of |log_2_ Ratio|≥1 for controlling false discovery rates.

|  | Total | Up-regulated | Down-regulated |
| --- | --- | --- | --- |
| CK30-VS-RD30 | 4,855 | 3,863(79.6%) | 992(20.4%) |
| CK80-VS-RD80 | 1,164 | 544(64.7%) | 620(35.3%) |
| CK170-VS-RD170 | 2,526 | 1,105(43.7%) | 1,421(56.3%) |
