# Supplementary material for: Transcriptome and Metabolome Analyses Provide Insights into the Occurrence of Peel Roughing Disorder on Satsuma Mandarin (Citrus unshiu Marc.) Fruit
Source: Front Plant Sci. 2017 Nov 7;8:1907. doi: 10.3389/fpls.2017.01907 (PMC5682035; doi:10.3389/fpls.2017.01907)
Supplement: Supplementary file 7 [file Table4.DOCX]

**Table S4 The number of differently expressed transcription factor during development of peel roughing disorder**

| **TF family** | **30DAFB** | | | **80DAFB** | | | **170DAFB** | | |
| --- | --- | --- | --- | --- | --- | --- | --- | --- | --- |
|  | **Total** | **Up** | **Down** | **Total** | **Up** | **Down** | **Total** | **Up** | **Down** |
| ABI3VP1 | 3 | 3 | 0 | 0 | 0 | 0 | 1 | 0 | 1 |
| AP2-EREBP | 14 | 10 | 4 | 8 | 3 | 5 | 8 | 3 | 5 |
| ARF | 5 | 5 | 0 | 0 | 0 | 0 | 5 | 2 | 3 |
| ARR-B | 6 | 6 | 0 | 0 | 0 | 0 | 0 | 0 | 0 |
| BBR/BPC | 1 | 1 | 0 | 0 | 0 | 0 | 0 | 0 | 0 |
| BES1 | 1 | 0 | 1 | 0 | 0 | 0 | 1 | 0 | 1 |
| bHLH | 32 | 25 | 7 | 10 | 6 | 4 | 21 | 11 | 10 |
| bZIP | 2 | 1 | 1 | 0 | 0 | 0 | 0 | 0 | 0 |
| C2C2-CO-like | 1 | 1 | 0 | 1 | 1 | 0 | 1 | 1 | 0 |
| C2C2-Dof | 6 | 5 | 1 | 2 | 0 | 2 | 1 | 1 | 0 |
| C2C2-GATA | 3 | 3 | 0 | 0 | 0 | 0 | 3 | 2 | 1 |
| C2C2-YABBY | 1 | 1 | 0 | 0 | 0 | 0 | 0 | 0 | 0 |
| C2H2 | 6 | 6 | 0 | 1 | 1 | 0 | 3 | 1 | 2 |
| C3H | 15 | 13 | 2 | 0 | 0 | 0 | 3 | 0 | 3 |
| CPP | 2 | 2 | 0 | 0 | 0 | 0 | 0 | 0 | 0 |
| DBP | 1 | 1 | 0 | 0 | 0 | 0 | 0 | 0 | 0 |
| E2F-DP | 3 | 0 | 3 | 0 | 0 | 0 | 0 | 0 | 0 |
| FAR1 | 3 | 3 | 0 | 0 | 0 | 0 | 0 | 0 | 0 |
| FHA | 6 | 6 | 0 | 0 | 0 | 0 | 0 | 0 | 0 |
| G2-like | 12 | 12 | 0 | 3 | 3 | 0 | 1 | 0 | 1 |
| GRAS | 10 | 8 | 2 | 2 | 2 | 0 | 6 | 1 | 5 |
| GRF | 8 | 8 | 0 | 1 | 1 | 0 | 3 | 3 | 0 |
| HB | 1 | 1 | 0 | 0 | 0 | 0 | 0 | 0 | 0 |
| HRT | 1 | 1 | 0 | 0 | 0 | 0 | 1 | 0 | 1 |
| HSF | 10 | 5 | 5 | 2 | 2 | 0 | 0 | 0 | 0 |
| LIM | 1 | 0 | 1 | 0 | 0 | 0 | 1 | 1 | 0 |
| LOB | 7 | 6 | 1 | 2 | 0 | 2 | 7 | 3 | 4 |
| MADS | 2 | 2 | 0 | 0 | 0 | 0 | 0 | 0 | 0 |
| mTERF | 9 | 9 | 0 | 0 | 0 | 0 | 3 | 1 | 2 |
| MYB | 40 | 35 | 5 | 15 | 10 | 5 | 16 | 3 | 13 |
| MYB-related | 25 | 20 | 5 | 11 | 7 | 4 | 14 | 3 | 11 |
| NAC | 12 | 6 | 6 | 7 | 3 | 4 | 13 | 4 | 9 |
| OFP | 3 | 3 | 0 | 1 | 1 | 0 | 2 | 0 | 2 |
| PLATZ | 2 | 2 | 0 | 0 | 0 | 0 | 0 | 0 | 0 |
| RWP-RK | 1 | 1 | 0 | 0 | 0 | 0 | 1 | 0 | 1 |
| Sigma70-like | 6 | 6 | 0 | 0 | 0 | 0 | 0 | 0 | 0 |
| TCP | 4 | 4 | 0 | 1 | 1 | 0 | 0 | 0 | 0 |
| Tify | 5 | 0 | 5 | 7 | 0 | 7 | 6 | 3 | 3 |
| Trihelix | 6 | 6 | 0 | 2 | 1 | 1 | 1 | 0 | 1 |
| TUB | 2 | 2 | 0 | 0 | 0 | 0 | 0 | 0 | 0 |
| WRKY | 15 | 9 | 6 | 7 | 0 | 7 | 16 | 0 | 16 |
| zf-HD | 3 | 3 | 0 | 0 | 0 | 0 | 0 | 0 | 0 |
| EIL | 0 | 0 | 0 | 0 | 0 | 0 | 2 | 0 | 2 |
| Total | 296 | 241 | 55 | 83 | 42 | 41 | 140 | 43 | 97 |
